# A room of her own: does the improvement of gender role attitude promote rural mothers’ entrepreneurship?—Evidence from China

**DOI:** 10.3389/fsoc.2025.1732202

**Published:** 2026-01-06

**Authors:** Kaichao Shao, Huijuan Ren, Peizhe Xu

**Affiliations:** 1 School of Finance and Accounting, Yellow River Conservancy Technical University, Kaifeng, Henan, China; 2 Department of Human Resources, Yellow River Conservancy Technical University, Kaifeng, Henan, China; 3 Institute of Food Economics, Nanjing University of Finance and Economics, Nanjing, Jiangsu, China

**Keywords:** entrepreneurship, gender role attitude, higher education, rural mothers, spillover effect

## Abstract

**Introduction:**

How does the change in gender role attitude reshape women’s entrepreneurship? This question holds significant practical implications for unleashing women’s economic potential and promoting inclusive economic development.

**Methods:**

Using data from the Chinese General Social Survey (CGSS), this study analyzes the impact of an improved gender role attitude on rural mothers’ entrepreneurship.

**Results:**

The results show that the improvement in gender role attitude increases the entrepreneurship rate of rural mothers by 4.89%. However, there are differences. The impact of an improved gender role attitude on rural mothers’ necessity-based entrepreneurship is 4% higher than that on opportunity-based entrepreneurship. Specifically, the improvement in gender role attitude has a significant positive effect on the entrepreneurship of rural mothers who have not completed higher education, while it shows the opposite effect on those who have completed higher education. Mechanism analysis reveals that family support and information access play a moderating role in the impact of gender role attitude on rural mothers’ entrepreneurship.

**Discussion:**

The study also finds that the entrepreneurial behavior of rural mothers has a spillover effect. Rural mothers provide more employment opportunities for other women through their entrepreneurial activities. Not only expands the research boundary of women’s entrepreneurship and the application scope of entrepreneurship theories, but also explores the internal mechanism through which gender role attitudes influence rural mothers’ entrepreneurship.

## 1 Introduction

A woman must have money and a room of her own if she is to write fiction. The lack of a private room forces women to write in the common sitting - room, subject to interruption.

——Virginia Woolf《A Room of One’s Own》

Women’s entrepreneurship plays a pivotal role in economic and social development (Díaz-García and Jiménez-Moreno, 2010; Ogundana et al., 2021; Rosa et al., 1996). According to statistics from the Global Entrepreneurship Monitor (GEM), the number of female entrepreneurs worldwide has continued to increase in recent years, which has played a positive role in promoting economic development and social progress. Particularly in developing countries, women’s entrepreneurship can not only increase employment and alleviate poverty but also enhance women’s social status and reduce gender discrimination (Rosca et al., 2020). Meanwhile, female entrepreneurs pay more attention to employees’ rights and interests, thereby promoting gender equality in the workplace (Sarpong et al., 2021). Therefore, promoting women’s entrepreneurship and unleashing their economic potential is of great significance for achieving inclusive economic and social development (Poggesi et al., 2016).

However, it cannot be ignored that there is a significant urban–rural gap in women’s entrepreneurship. Urban women have higher educational attainment and a more favorable entrepreneurial environment, so their entrepreneurship rate is significantly higher than that of rural women. In contrast, rural women, especially rural mothers, are constrained by social norms and family division of labor, and their entrepreneurial activities face a more severe “motherhood penalty” (Mahmud et al., 2017).

Gender role attitude is a key factor influencing the entrepreneurial enthusiasm of rural mothers (Correll et al., 2007). In developing countries, rural mothers are primarily evaluated from the perspectives of childbearing and family responsibilities, rather than from the dimensions of productive contributions and income generation. Social norms and the division of household lab or restrict the entrepreneurial behaviors of rural mothers (Kobeissi, 2010), which imposes significant constraints on rural mothers when they attempt to take on roles beyond that of a housewife (Chordiya, 2013; Nguyen et al., 2014).

On the one hand, barriers from traditional culture and gender-based division of labor (Cooke and Xiao, 2021). In the division of labor within families, childcare and household chores are regarded as the primary responsibilities of rural mothers, and women are expected to prioritize family over career (Liu, 2013). As a result, rural mothers take on nearly all childcare and household tasks, which significantly limits both the time they can devote to entrepreneurship and their enthusiasm for it (Wang et al., 2019).

On the other hand, lack of sufficient economic (gender) empowerment. Amartya Sen (2005) argued that poverty is not merely low income, but the deprivation of basic rights. Bhatia and Singh (2019) found through their research that insufficient economic empowerment severely restricts women’s economic autonomy and also hinders their ability to access opportunities. Additionally, the lack of professional skills and entrepreneurial training makes it difficult for rural mothers to acquire the necessary business skills and knowledge (Koku, 2015; Zhang et al., 2020). The dual shortcomings of insufficient economic empowerment and lack of skills mean that even if rural women have entrepreneurial aspirations, they find it hard to realize them (Mahmud et al., 2017).

Obviously, gender role attitude is a crucial factor influencing rural mothers’ entrepreneurship. Especially in developing countries, activating the entrepreneurial vitality of rural mothers plays a crucial role in inclusive economic development. As one of the world’s largest developing countries, China boasts a substantial scale of rural mothers (with over 200 million rural mothers). Supporting the entrepreneurial activities of rural mothers is therefore vital for promoting the development of China’s rural economy. With the deepening of China’s reform and opening-up, and the continuous implementation of policies such as the compulsory education system and women’s empowerment initiatives, the gender role attitude of rural mothers has undergone tremendous changes. Against this background, this study focuses on the following two research questions. First, whether the shift in gender role attitudes has reshaped rural mothers’ entrepreneurial behaviors. Second, what specific internal mechanisms mediate or moderate the impact of gender role attitude changes on their entrepreneurial engagement and outcomes.

To explore these questions, this study uses data from the Chinese General Social Survey (CGSS 2010–2023) and employs a Probit model to investigate the impact of improved gender role attitude on the entrepreneurship of rural mothers in China. Furthermore, it analyzes the heterogeneity and mechanisms of rural mothers’ entrepreneurial behaviors. The marginal contributions of this study are first analyzing the entrepreneurial behaviors of the rural mother group from the perspective of changes in gender role attitude to expand the research perspective on women’s entrepreneurship, second pioneering the construction of an analytical framework for female entrepreneurs based on entrepreneurship theory to extend the application boundary of entrepreneurship theory, and third examining the role mechanisms of family support and information effect in the impact of improved gender role attitude on rural mothers’ entrepreneurship, verifying the spillover effect of rural mothers’ entrepreneurial behaviors, and deconstructing the internal mechanisms of rural mothers’ entrepreneurial behaviors.

## 2 Theoretical analysis and research hypotheses

Gender role attitude influence rural mothers’ recombination of production factors and identification of entrepreneurial opportunities in the entrepreneurial process. Based on the Innovation and Entrepreneurship Theory, this study constructs an analytical framework to explore how gender role attitudes affect rural mothers’ entrepreneurship. On the one hand, drawing on the resource integration and opportunity identification dimensions of the Innovation and Entrepreneurship Theory, it theoretically analyzes the direct effects of gender role attitudes on rural mothers’ entrepreneurial activities. On the other hand, it takes family support and information acquisition as moderating variables to examine the moderating mechanisms through which gender role attitudes impact rural mothers’ entrepreneurship.

### 2.1 Gender role attitude and rural mothers’ entrepreneurship

The innovation and entrepreneurship theory holds that entrepreneurship is the driving force behind economic development, and entrepreneurs achieve the “new combination of production factors” through “creative destruction” (Schumpeter, 1934). However, a growing body of research shows that there are significant gender differences in entrepreneurship (Akehurst et al., 2012). For example, in terms of entrepreneurial motivation, women’s entrepreneurship is more driven by factors such as insufficient income, unemployment, and underemployment (Birley, 1987; DeMartino and Barbato, 2003). In terms of entrepreneurial characteristics, female entrepreneurs are more averse to market risks than male entrepreneurs (Rey-Martí et al., 2015), and the scale of enterprises managed by female entrepreneurs is smaller (Warren-Smith, 2012). Nevertheless, an increasing number of studies have found that the gender differences in entrepreneurship do not stem from women’s own lack of ability, but from the constraints of social systems, such as gender attitudes and gender role inconsistency. Therefore, gender role attitude has a significant impact on women’s entrepreneurship, especially on rural mother entrepreneurs (Foss et al., 2019; Maden, 2015).

Among female entrepreneurs, rural mothers are a special group. They not only face practical dilemmas of family care but also are restricted by the objective environment of relatively underdeveloped rural areas. Within families, there are deeply rooted concepts of family division of labor in rural areas, such as “valuing sons over daughters” and “wives being subordinate to husbands,” which have long kept rural mothers in a subordinate position. They not only bear most of the household chores but also rarely have the right to voice opinions on family decisions (Dildar, 2015; Kamphoff, 2010). In terms of the external environment, weak infrastructure, inconvenient transportation, and low network coverage in rural areas have constrained the entrepreneurial behaviors of rural mothers (Poon et al., 2012). Additionally, there are obvious gender discrimination phenomena in fields such as bank loan approval and the allocation of entrepreneurial training resources (Constantinidis et al., 2006; Harrison and Mason, 2007). Therefore, gender bias has hindered the economic empowerment of rural mothers and widened the gender gap in the entrepreneurial process (Agyire-Tettey et al., 2018). In contrast, the improvement of gender role attitude plays a promoting role in the entrepreneurship of rural mothers (Wang et al., 2019).

From the perspective of individual development, the improvement of gender role attitude provides a practical path for the economic empowerment of rural mothers. As rural mothers’ gender role attitude improves, more and more of them break through the dual dilemmas of traditional gender division of labor and social gender bias, proactively participate in economic activities, and acquire more resources. This creates the possibility for the “recombination of production factors” in the entrepreneurial process (Corrigall and Konrad, 2007; Eccles, 1987; Uunk, 2015). Meanwhile, based on their own life experiences and market observations, rural mothers actively identify new entrepreneurial opportunities and engage in entrepreneurial activities (Scarborough et al., 2019). Therefore, from the perspective of entrepreneurship, against the backdrop of improved gender role attitude, rural mothers break free from the constraints of traditional cognitive frameworks. By identifying entrepreneurial opportunities and recombining production factors, they unleash the human capital and innovative spirit inherent in entrepreneurs (Bolzendahl and Myers, 2004).

From the perspective of social development, the improvement of gender role attitude is conducive to enhancing the entrepreneurial capabilities of rural mothers. In traditional rural societies, rural mothers have often faced numerous restrictions and unfair treatment (Archer, 1996). Due to the traditional gender role attitude that men are more suitable for entrepreneurship, rural mothers have long been in a marginalized position in the field of entrepreneurship (Merrett and Gruidl, 2000). However, with the continuous improvement and advancement of social perceptions toward gender, such biases are gradually diminishing (Krook and True, 2012). The improvement of gender role attitude means that society pays more attention to the development needs of rural mothers and provides corresponding support and resources. Governments, social organizations, and enterprises have begun to attach importance to and promote the entrepreneurial development of rural mothers (Grown et al., 2016). Based on the above analysis, we propose the following hypothesis:

*H1*: The improvement of gender role attitude promotes the entrepreneurship of rural mothers.

### 2.2 The importance of family gender role attitude

The impact of family gender role attitude on rural women cannot be ignored, as it directly affects their family status and entrepreneurial behaviors (Edelman et al., 2016). In traditional rural families, husbands usually occupy a dominant position, and their gender role attitude exerts a profound influence on their wives (Jennings and Brush, 2013). If husbands hold traditional notions such as “valuing sons over daughters” and “women should prioritize family,” they tend to require their wives to take on all household chores and the responsibility of caring for children. This prevents wives from accessing skills training and participating in economic activities (Zhang and Zhou, 2019). In such an environment, for wives to start their own businesses, they must obtain their husbands’ approval and support. Conversely, if husbands advocate “gender equality” and encourage their wives to seek development opportunities outside the family, women are more likely to engage in entrepreneurial activities (Henry et al., 2016). It is evident that husbands’ gender role attitude is one of the key factors influencing whether rural mothers can start businesses. In China, as the younger generation receives more modern education, the perception of gender roles within families is gradually changing, which provides family support for rural mothers’ entrepreneurship.

Parents’ educational level also exerts a significant influence on rural mothers’ gender perception (Kane, 2006). In traditional rural societies, restricted by factors such as limited economic conditions, cultural traditions, and insufficient educational opportunities, many women lack awareness of their own rights and potential (Dhar et al., 2019). Moreover, parents’ educational level is often a key factor affecting women’s gender perception (Pyne, 2016). The higher the parents’ educational level, the greater emphasis they place on their children’s education (Perales et al., 2021). Additionally, they begin to recognize that daughters possess the same developmental capabilities as sons, thus providing more educational opportunities for their daughters (Biblarz and Stacey, 2010). After receiving education, rural mothers also come to realize that they have equal rights and opportunities to engage in entrepreneurship (England, 2010). Furthermore, the higher the parents’ educational level, the more they encourage their daughters to pursue economic independence, guide them to establish a correct gender role attitude, and provide support and assistance (Halimi et al., 2016; Hess et al., 2014). Based on the above analysis, we propose the following hypothesis:

*H2*: Husbands' gender role attitude and parents' educational level play a moderating role in rural mothers' gender role attitude, which in turn affects their entrepreneurial behaviors.

### 2.3 The importance of information dissemination

The development and dissemination of information technology (IT) contribute to advancing the concept of gender equality (Summers et al., 2020; Wajcman, 2010). The wealth of gender equality related content available on the internet gives women access to feminist perspectives and concepts of gender equity (Gnambs, 2021). Meanwhile, IT offers more opportunities for informal education, supporting rural women in self-directed learning and the enhancement of their skill sets (Zhou et al., 2020). Furthermore, IT provides women with additional platforms to voice their demands and viewpoints. They can disseminate feminist ideas through podcasts, social media accounts, and other channels. This holds significant importance for improving rural mothers’ awareness of gender equality.

Information technology (IT) also provides convenience for rural mothers’ entrepreneurship (Shao et al., 2022). Firstly, the internet enables rural mothers to access entrepreneurship related knowledge more conveniently (Barnett et al., 2019; Georgellis and Wall, 2005). Secondly, IT lowers the threshold for entrepreneurship (Janson and Wrycza, 1999). Rural mothers can engage in online entrepreneurship through online stores, live streaming, and other forms. Thirdly, IT breaks down geographical barriers in the entrepreneurial market, allowing rural mothers’ target markets to no longer be limited to their local areas (Barnett et al., 2019; Rashid, 2016). Finally, IT offers more flexible entrepreneurial models. By leveraging the internet for entrepreneurship, rural mothers can take care of their families while running their businesses online. Based on the above analysis, we propose the following hypothesis:

*H3*: The development of information technology (IT) plays a moderating role in rural mothers' gender role attitude, which in turn affects their entrepreneurial behaviors.

## 3 Data, variables, and model

### 3.1 Data

The relevant data in this study are mainly derived from the Chinese General Social Survey (CGSS). As China’s earliest national-level, comprehensive, and continuous academic survey project, CGSS is jointly implemented by the National Survey Research Center at Renmin University of China and more than 40 universities and research institutions across China. Since 2003, CGSS has conducted more than 10 rounds of surveys. Considering the availability of indicators, this study uses data from 9 waves of CGSS, namely CGSS 2010, 2012, 2013, 2015, 2017, 2018, 2021 and 2023.

To ensure the reliability of the research results, the data were processed as follows: (1) Excluding unmarried and childless women, only married and childbearing rural women were retained; (2) Considering potential biases caused by cross-regional mobility of the rural population, rural women whose household registration was inconsistent with their place of residence were excluded; (3) Excluding rural women over 60 years old and under 18 years old, with the sample limited to rural women aged 18–60; (4) Excluding college students and individuals with loss of labor capacity. Finally, the number of valid samples of rural mothers meeting the criteria was 10,415.

### 3.2 Variables

#### 3.2.1 Dependent variable

Rural Mothers’ Entrepreneurship. Based on the question in the questionnaire asking respondents “*Whether there exist entrepreneurial activities*,” a dummy variable was created in this study. If the respondent answered “*The existence of entrepreneurial activities*,” the dummy variable was assigned a value of 1, indicating an entrepreneurial sample; otherwise, it was assigned a value of 0.

Kalemci Tuzun and Araz Takay (2017) found that female entrepreneurs in developing countries include two types: subsistence entrepreneurship and opportunity entrepreneurship. Subsistence entrepreneurship refers to entrepreneurial activities carried out to meet basic livelihood needs, while opportunity entrepreneurship refers to proactive and innovative business activities conducted by leveraging market demands. To further analyze the impact of improved gender role attitude on different types of entrepreneurship among rural mothers, another dummy variable was created based on the questionnaire question asking respondents “*How many employees do you have?*.” If the respondent answered that they had more than one employee, the dummy variable was assigned a value of 1, representing an opportunity entrepreneurship sample. if the respondent answered that they had zero employees, the dummy variable was assigned a value of 0, representing a subsistence entrepreneurship sample.

#### 3.2.2 Independent variable

Gender role attitude. Gender role attitude refers to an individual’s views on gender roles and perception of differences between the two sexes. In China, traditional Confucianism holds that men should take on the family’s economic responsibilities, while women should be responsible for caring for the family and children (Tatli et al., 2017). Although the social status of Chinese women has improved in recent years, stereotypes about the roles of men and women still persist due to the influence of Confucianism.

Based on the Gender Role Attitude Scale (GRAS) (Garcia-Cueto et al., 2015; Halimi et al., 2018; Celebi Cakiroglu and Harmanci Seren, 2022), this study developed a simplified gender role attitude measurement tool from three core dimensions: cognition, emotion, and behavioral. Specifically, the cognitive dimension corresponds to the item “*Men are inherently more capable than women*,” the emotional dimension corresponds to “*Marriage is better than career success for women*,” and the behavioral tendency dimension corresponds to “*Couples should not share housework equally*.” Each item of the scale adopts a 5-point Likert scoring method, with scores ranging from 0 to 4. The total score of the scale is obtained by summing the scores of items across all dimensions, where a higher total score indicates a stronger acceptance of gender equality attitudes. Details are shown in Table 1.

**Table 1:** Gender role attitude scale.

Dimensions	Question	Strongly agree	Agree	Neutral	Disagree	Strongly disagree
Cognition	*Men are inherently more capable than women*	0	1	2	3	4
Emotion	*Marriage is better than career success for women*	0	1	2	3	4
Behavioral	*Couples should not share housework equally*	0	1	2	3	4

To verify the robustness of the benchmark regression results of the GRAS, this study further constructs an evaluation indicator based on the question “*Whether one agrees that men should prioritize their careers while women prioritize family* (GRAS1).” Respondents’ responses include: Strongly agree, Agree, Neutral, Disagree, and Strongly disagree, which are assigned values of 0 to 4, respectively (Table 2).

**Table 2:** Variable definitions, measurement items, and coding.

Variable	Definition	Measurement item	Coding range
Entrepreneurship	Entrepreneurial status	Whether there is entrepreneurial activity.	Yes = 1, No = 0
GRAS	Gender Role Attitude Scale	Gender Role Attitude Scale	0–12
GRAS1	Gender role attitude	Men focus on careers, women on family	0–4
Age	Age	What is your date of birth?	18–60
Religion	Religious belief	What is your religious belief?	Yes = 1, No = 0
Years of education	Years of education	What is your current education level?	0–19
Household income	Annual household income	What was your total household income last year?	300–1,500,000
Political identity	Political identity	What is your current political identity?	Yes = 1, No = 0
Health	Health status	How would you describe your current health status?	0–4
Information sources	Information acquisition channels	What are your main sources of information?	1–6
Real Estate	Number of real estates	How many properties do you own in your household?	1–14
Car	Number of cars owned	Does your household own a car?	0–1
Number of children	Number of children raised	How many children have you raised?	1–8
Spouse Education	Spouse’s years of education	What is the education level of your husband or father?	0–19
Father Education	Father’s years of education	What is the education level of your father?	0–16
Mother Education	Mother’s years of education	What is the education level of your mother?	0–16
Spillover effect	Women’s employment status	Should female employees be laid off first?	0–4

#### 3.2.3 Control variables

Firstly, at the individual level, we drew on Shao et al. (2023) and included rural mothers’ age, religious belief, political identity, education level, number of children, and health status as control variables.

Age was calculated based on the respondent’s answer to the question: “*What is your date of birth?*”

Religious belief was measured using a dummy variable created from the question: *“What is your religious belief?”* Yes, is assigned a value of 1, and no a value of 0.

Political identity was measured using a dummy variable created from the question: “*What is your current political identity?*” Yes, is assigned a value of 1, and no a value of 0.

Education level was measured using a variable created from the question: “*What is your current education level?*” Specific assignments were as follows: primary school (6 years), junior high school (9 years), senior high school (12 years), junior college (15 years), undergraduate (16 years), master’s degree (18 years), and doctoral degree (21 years).

The number of children was measured using a variable created from the question: “*How many children have you raised?*”

Health status was measured using a dummy variable created from the question: “*How would you describe your current health status?*” Respondents’ answers (*“very unhealthy,” “somewhat unhealthy,” “average health,” “relatively healthy,” “very healthy”*) were assigned values of 0 to 4, respectively.

Secondly, at the household level, household income, household property status (housing), and ownership of a vehicle (car) were included as household-level control variables for rural mothers’ entrepreneurship.

Household income was measured using a variable created from the question: “*What was your total household income last year?*”

Household property status (housing) was measured using a variable created from the question: “*How many properties (houses) do you own in your household?*”

Ownership of a vehicle (car) was measured using a dummy variable created from the question: “*Does your household own a car?*” Yes, is assigned a value of 1, and no a value of 0.

#### 3.2.4 Moderating variables

The moderating variables include family gender role attitude, information acquisition, and the spillover effect of entrepreneurship.

Family gender role attitude. This variable was proxied by the education level of the respondent’s husband or father. A variable was created based on the respondent’s answer to the question: “*What is the education level of your husband or father?*” Specific assignments were as follows: primary school (6 years), junior high school (9 years), senior high school (12 years), junior college (15 years), undergraduate (16 years), master’s degree (18 years), and doctoral degree (21 years).

Information Acquisition. A variable was created based on the questionnaire question asking respondents: “*What are your main sources of information?*” Respondents’ answers (*newspapers, magazines, radio programs, television, the Internet, and mobile phones*) were assigned values of 1 to 6, respectively.

Spillover Effect of Entrepreneurship. A variable was created based on the questionnaire question asking respondents: “*In times of economic downturn, should female employees be laid off first?*” Respondents’ answers (*strongly agree, somewhat agree, neutral, somewhat disagree, strongly disagree*) were assigned values of 0 to 4, respectively.

### 3.3 Model

To verify the impact of improved gender role attitude on rural women’s entrepreneurship, this study constructs a Probit model for empirical analysis based on the characteristic that the dependent variable is a binary discrete variable. In the model, the dependent variable is the entrepreneurial status of rural women, the independent variable is gender role attitude, and a series of control variables are also included. The specific Equation 1 is as follows:


$$Entrepreneurship_{i}=\beta_{0}+\beta_{1}GRAS+\beta_{2}\sum control_{i}+\mu_{i}+year+\varepsilon_{i}$$ (1)

Among them, *β* denotes the coefficient to be estimated, year represents the year dummy variable, *μ* denotes the provincial-level regional dummy variable, *i* stands for the individual, and *ε* is the random error term. *Entrepreneurship* is the dummy variable for rural mothers’ entrepreneurship. GRAS refers to the gender role attitude of individual samples. Control represents the control variables, including individual characteristics and household characteristics. *u_i_* and *year_i_* denote the provincial-level regional fixed effect and time fixed effect, respectively.

To examine the moderating mechanism of gender role attitude on rural mothers’ entrepreneurship, we introduce the interaction terms between three variables (family gender role attitude, the role of information, and the spillover effect of entrepreneurship) and the independent variable into the analysis based on Equation 1 for verification. The Equation 2 design is as follows:


$$\begin{array}{l} Entrepreneurship_{i}=\beta_{0}+\beta_{1}GRAS_{i}+\beta_{2}Z_{i}+\beta_{3}GRAS_{i}\times Z_{i}+\\ \beta_{4}\sum control_{i}+\mu_{i}+year+\varepsilon_{i} \end{array}$$ (2)

Where denotes the moderating variables, which are family gender role attitude, information acquisition channels, and the spillover effect of entrepreneurship, respectively. Represents the interaction term between gender role attitude (the independent variable) and the moderating variables. These interaction terms, respectively, reflect the moderating roles of family gender role attitude, information acquisition channels, and the spillover effect of entrepreneurship on the impact of individual gender role attitude on rural mothers’ entrepreneurship.

### 3.4 Descriptive statistics

**Figure 1:**
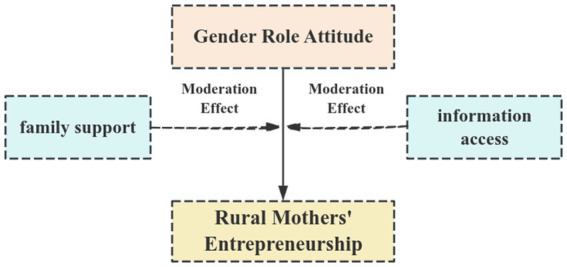
Direct effects and moderating effects of gender role attitudes on rural mothers’ entrepreneurship.

Table 3 presents the descriptive statistics of the variables. In terms of rural mothers’ entrepreneurship, the mean value is 0.1047 with a standard deviation of 0.3062, indicating that the proportion of rural mothers who engage in entrepreneurship is relatively low, accounting for only 7.05% of the total sample. For the GRAS, the mean value and standard deviation are 6.5398 and 2.2751 respectively, suggesting that rural mothers’ acceptance of gender equality attitudes is relatively low with significant variability. The mean value and standard deviation of GRAS1 are 1.4388 and 1.2075 respectively, which further confirms the view that rural mothers’ gender equality attitudes are relatively weak (Figure 1).

**Table 3:** Descriptive statistics.

Variable	Obs.	Mean	Std. dev.	Min	Max
Rural Mother entrepreneurship	10,415	0.1074	0.3096	0	1
GRAS	10,415	6.5398	2.2751	0	12
GRAS1	10,415	1.4388	1.2075	0	4
Age	10,415	44.0593	9.8319	18	60
Religion	10,415	0.1102	0.3131	0	1
Years of education	10,415	6.9363	3.7721	0	19
Household income	10,415	42747.82	54606.61	300	1,500,000
Political identity	10,415	0.0361	0.1867	0	1
Health	10,415	2.5473	1.1056	0	4
Information sources	10,415	4.5135	0.9056	1	6
Real Estate	10,415	1.1138	0.4981	0	14
Car	10,415	0.2035	0.4026	0	1
Number of children	10,415	1.8625	0.8068	1	8
Spouse Education	10,415	8.1976	3.1961	0	19
Father Education	10,415	4.0246	3.9798	0	16
Mother Education	10,415	2.1258	3.2758	0	16
Spillover effect	10,415	2.8747	1.0256	0	4

Regarding individual characteristics, the average age of rural women in the sample is 44 years old, with an average of 6.9 years of education and an overall good health status. In terms of household characteristics, the average annual household income is 42,747.82 yuan, the average number of children raised is 2, and most households own one piece of real estate, while only 18% of households own a car. Correspondingly, the average years of education of spouses (husbands) is 8.1 years, meaning that the average educational level of males is higher than that of females.

**Figure 2:**
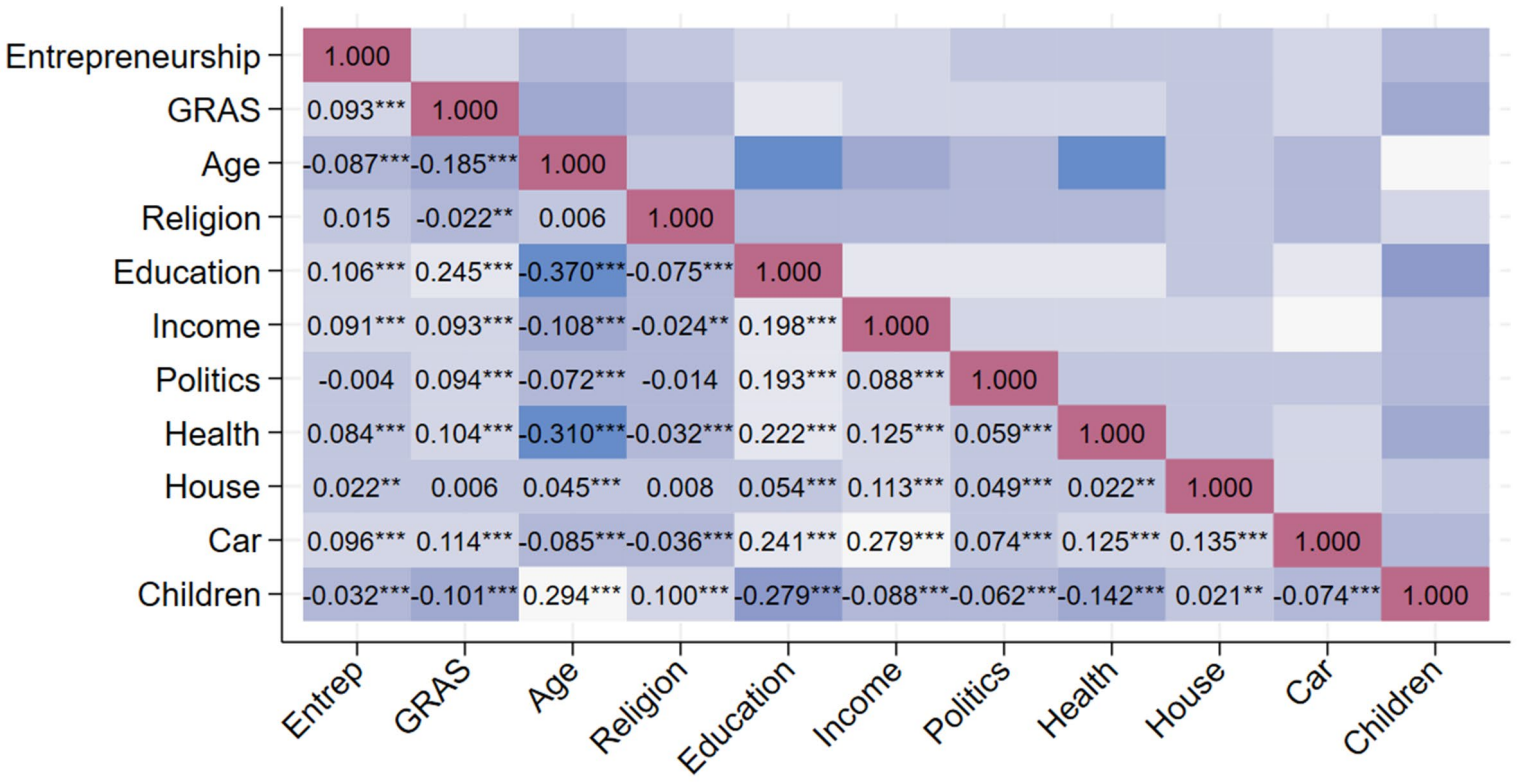
Heatmap of correlation test.

To ensure the absence of multicollinearity among variables, we conducted a Pearson correlation test. The test results show that the maximum correlation coefficient is 0.37, which is lower than the threshold of 0.8. Therefore, there is no multicollinearity issue (see Figure 2).

## 4 Empirical analysis

### 4.1 Baseline regression

Table 4 presents the stepwise regression results of the Probit model. Model (1) shows that without including control variables or controlling for time and regional fixed effects, the coefficient of GRAS is 0.0685, which is statistically significant at the 1% level. Model (2) indicates that when control variables are not included but time and regional fixed effects are controlled for, the coefficient of GRAS is 0.0711, also significant at the 1% level. Model (3) reveals that with control variables included but no control for time and regional fixed effects, the coefficient of GRAS is 0.0447, significant at the 1% level. Model (4) demonstrates that when both control variables and time and regional fixed effects are incorporated, the coefficient of GRAS is 0.0489, still statistically significant at the 1% level. These results verify Hypothesis 1 of this study: the improvement of gender attitudes promotes rural mothers’ entrepreneurship. Specifically, for every 1% increase in Gender role attitudes, the probability of rural mothers engaging in entrepreneurship increases by 4.89%.

**Table 4:** Baseline regression results.

Variables	(1)	(2)	(3)	(4)
GRAS	0.0685***	0.0711***	0.0447***	0.0489***
	(0.0072)	(0.0073)	(0.0075)	(0.0076)
Age			−0.0063***	−0.0042**
			(0.00188)	(0.0021)
Religion			0.1332**	0.1081*
			(0.0526)	(0.0584)
Years of education			0.0274***	0.0309***
			(0.0054)	(0.0057)
Household income			0.0119***	0.0115***
			(0.0027)	(0.0029)
Political identity			−0.2926***	−0.2874***
			(0.0984)	(0.0986)
Health			0.0772***	0.0812***
			(0.0169)	(0.0175)
Real Estate			0.0193	0.0239
			(0.0331)	(0.0338)
Car			0.2003***	0.3015***
			(0.0412)	(0.0463)
Number of children			0.0215	0.0038
			(0.0232)	(0.0249)
_cons	−1.703***	−2.028***	−1.846***	−2.431***
	(0.0526)	(0.368)	(0.134)	(0.401)
Region	No	Yes	No	Yes
Year	No	Yes	No	Yes
N	10,415	10,415	10,415	10,415
Pseudo R2	0.0127	0.0318	0.0409	0.0621

① ***, **, and * signify that the estimation findings are significant at the 1, 5, and 10% levels, respectively. ② The robust standard errors are enclosed in parentheses.

Furthermore, by examining the regression results of the control variables, it can be found that the age and political identity of rural mothers are significantly negatively correlated with their entrepreneurship. This indicates that rural mothers should start their businesses as early as possible, as older age is less conducive to their entrepreneurial activities.

In contrast, the religious belief, education level, health status, household income, and vehicle ownership of rural mothers exhibit a significant positive correlation with their entrepreneurship. Specifically, this indicates that rural mothers with religious beliefs are more inclined to engage in entrepreneurial activities. Better family economic conditions are more conducive to rural mothers’ entrepreneurship, and rural mothers in better health have a stronger intention to start businesses.

### 4.2 Robustness tests

To ensure the reliability of the baseline regression results, the following robustness tests were conducted in this study.

#### 4.2.1 Alternative regression method

Although this study used the Probit model to estimate the impact of gender role attitude on rural mothers’ entrepreneurship, most of the independent variables and control variables are continuous in terms of data characteristics. Therefore, we re-estimated the results using the OLS model. Table 5 presents the regression results of the OLS model. Models (1) to (4) show that the coefficients corresponding to gender role attitude are 0.0127, 0.0131, 0.0084 and 0.0091 respectively, all of which have passed the significance test. This indicates that the improvement of gender role attitude promotes the entrepreneurial behavior of rural mothers, which is basically consistent with the results of the baseline regression.

**Table 5:** Robustness test - replacement of regression method.

Variables	(1)	(2)	(3)	(4)
GRAS	0.0127***	0.0131***	0.0084***	0.0091***
	(0.0013)	(0.0013)	(0.0013)	(0.0013)
Age			−0.0012***	−0.0078**
			(0.0003)	(0.0003)
Religion			0.0229**	0.0187*
			(0.0101)	(0.0109)
Years of education			0.0044***	0.0049***
			(0.0008)	(0.0009)
Household income			0.0303***	0.0293***
			(0.0061)	(0.0058)
Political identity			−0.0561***	−0.0531***
			(0.0168)	(0.0166)
Health			0.0125***	0.0133***
			(0.0027)	(0.0028)
Real Estate			0.0041	0.0053
			(0.0068)	(0.0067)
Car			0.0431***	0.0589***
			(0.0091)	(0.0097)
Number of children			0.0042	0.0015
			(0.0041)	(0.0424)
_cons	0.0243***	0.0289***	0.0779***	0.0969*
	(0.00877)	(0.0072)	(0.0232)	(0.0524)
Region	No	Yes	No	Yes
Year	No	Yes	No	Yes
N	10,415	10,415	10,415	10,415
R2	0.009	0.022	0.029	0.044

① ***, **, and * signify that the estimation findings are significant at the 1, 5, and 10% levels, respectively. ② The robust standard errors are enclosed in parentheses.

#### 4.2.2 Exclusion of specific values method

Exclusion of specific regions. Municipalities directly under the Central Government and autonomous regions are two special types of provincial-level administrative units in China, differing from ordinary provincial-level units in terms of economy and influence. Given the particularity of autonomous regions and municipalities directly under the Central Government, we excluded samples from Shanghai, Beijing, Tianjin, Chongqing, Inner Mongolia, Guangxi, and other such regions. Exclusion of specific age groups. Wang et al. (2019) argued that rural female entrepreneurs exhibit an inverted “U”-shaped age characteristic, meaning that the probability of their entrepreneurship tends to decrease as age increases. To ensure that the rural mothers in the sample still have entrepreneurial vitality, we excluded women over the age of 55 from the sample. As shown in Table 6, the test results indicate that gender role attitude is significantly positively correlated with rural mothers’ entrepreneurship.

**Table 6:** Robustness test - exclusion of specific samples.

Variables	(1)	(2)	(3)	(4)
GRAS	0.0733***	0.0756***	0.0481***	0.0519***
	(0.0077)	(0.0078)	(0.0081)	(0.0081)
Age			−0.0065***	−0.0048**
			(0.0021)	(0.0021)
Religion			0.1514***	0.1285**
			(0.0577)	(0.0607)
Years of education			0.0275***	0.0306***
			(0.0059)	(0.0061)
Household income			0.0113***	0.0101***
			(0.0028)	(0.0031)
Political identity			−0.2744***	−0.2767***
			(0.1062)	(0.1064)
Health			0.0852***	0.0937***
			(0.0181)	(0.0188)
Real Estate			0.0419	0.0492
			(0.0372)	(0.0377)
Car			0.2394***	0.3376***
			(0.0438)	(0.0495)
Number of children			0.0179	−0.000915
			(0.0256)	(0.0269)
_cons	1.723***	1.976***	1.902***	2.137***
	(0.0562)	(0.107)	(0.145)	(0.168)
Region	No	Yes	No	Yes
Year	No	Yes	No	Yes
N	9,100	9,092	9,100	9,092
Pseudo R2	0.0143	0.0322	0.0463	0.0668

① ***, **, and * signify that the estimation findings are significant at the 1, 5, and 10% levels, respectively. ② The robust standard errors are enclosed in parentheses.

#### 4.2.3 Alternative explanatory variables

In the benchmark regression, this study uses the GRAS scale as the core explanatory variable. To ensure the reliability and robustness of this measurement, this study replaces the GRAS scale with the evaluation of “*Whether one agrees that men should prioritize their careers while women prioritize family*” (GRAS1) and re-estimates the impact of gender attitudes on rural mothers’ entrepreneurship. Table 7 shows that Gender role attitude exhibit a significantly positive correlation with rural mothers’ entrepreneurship. This further confirms the robustness of the benchmark regression results.

**Table 7:** Robustness test - variable replacement method.

Variables	(1)	(2)	(3)	(4)
GRAS1	0.1085***	0.1208***	0.0604***	0.0791***
	(0.0135)	(0.0141)	(0.0143)	(0.0147)
Age			−0.0068***	−0.0045**
			(0.0019)	(0.0021)
Religion			0.1375***	0.1115*
			(0.0527)	(0.0585)
Years of education			0.0291***	0.0325***
			(0.0054)	(0.0057)
Household income			0.0118***	0.0114***
			(0.0026)	(0.0028)
Political identity			−0.2866***	−0.2865***
			(0.0981)	(0.0982)
Health			0.0775***	0.0823***
			(0.0169)	(0.0176)
Real Estate			0.0221	0.0269
			(0.0335)	(0.0338)
Car			0.1956***	0.2996***
			(0.0413)	(0.0464)
Number of children			0.0255	0.0078
			(0.0232)	(0.0249)
Region	No	Yes	No	Yes
Year	No	Yes	No	Yes
_cons	1.406***	1.687***	1.636***	2.209***
	(0.0271)	(0.359)	(0.125)	(0.394)
N	10,415	10,406	10,415	10,406

① ***, **, and * signify that the estimation findings are significant at the 1, 5, and 10% levels, respectively. ② The robust standard errors are enclosed in parentheses.

#### 4.2.4 Instrumental variable method

This study may face endogeneity issues caused by reverse causality, omitted variables, and other factors, leading to biases in the estimation results. To address this, the instrumental variable method is adopted to correct such biases. In this paper, the education level of rural mothers’ biological mothers (abbreviated as “maternal education level”) is selected as the instrumental variable for rural mothers’ gender role attitude. From the perspective of correlation, the mother’s education level has a significant correlation with the gender attitudes formed by her daughter. Mothers with a higher education level are more likely to pass on equal gender attitudes to their daughters (Endendijk et al., 2013). Therefore, the mother’s education level can influence the daughter’s gender attitudes, satisfying the correlation condition of instrumental variables. From the perspective of exogeneity, the mother’s education level is irrelevant to the daughter’s entrepreneurial decision-making and does not have a direct impact on the daughter’s entrepreneurship. Thus, the mother’s education level meets the exogeneity condition of instrumental variables.

Thirdly, from the perspective of independence: The mother’s education level affects the daughter’s entrepreneurial decision-making indirectly only through the channel of influencing the daughter’s gender attitudes, rather than having a direct impact on the daughter’s entrepreneurial behavior. At the same time, the mother’s education level is also irrelevant to other unobservable factors (i.e., the error term) that affect the daughter’s entrepreneurship. In summary, the mother’s education level can influence the daughter’s gender attitudes but does not affect the daughter’s entrepreneurship. Using the mother’s education level as an instrumental variable can effectively solve the endogeneity problem.

Table 8 presents the results of the instrumental variable (IV) regression. The results show that the Durbin–Wu–Hausman test statistics are 5.179 and 3.712, which reject the null hypothesis that “Gender role attitude are exogenous variables” at the 5 and 1% significance levels, respectively, confirming the existence of endogeneity. The weak instrumental variable test indicates that the Cragg-Donald Wald F statistics are 28.541 and 29.786, respectively, far exceeding the Stock-Yogo critical value of 16.38. Meanwhile, the t-values of the coefficients of the instrumental variables on the endogenous variable (Gender role attitude) are 2.871 and 2.786, suggesting a strong correlation between the instrumental variables and the endogenous variable, which effectively eliminates the bias caused by weak instrumental variables. The IV regression results show that the estimated coefficients of Gender role attitude in Model (1) and Model (2) are 0.0551 and 0.1025, respectively, both passing the significance test. This result is consistent with the conclusion of the benchmark regression, further verifying that the improvement of Gender role attitude promotes rural mothers’ entrepreneurship.

**Table 8:** Robustness test - instrumental variable method.

Variables	(1)	(2)
GRAS	0.0551*	
	(0.0294)	
GRAS1		0.1025*
		(0.0548)
Age	−0.0129	−0.0601
	(0.0106)	(0.0837)
Religion	0.0239**	0.0288**
	(0.0108)	(0.0118)
Years of education	0.0036***	−0.0319*
	(0.0011)	(0.0185)
Household income	0.0093***	0.0096***
	0.0036***	−0.0319*
Political identity	−0.0794**	−0.0739**
	(0.0316)	(0.0291)
Health	0.0112***	0.0985**
	(0.0364)	(0.0415)
Real Estate	0.0852	0.0128*
	(0.0716)	(0.0076)
Car	0.0374**	0.0254
	(0.0172)	(0.0229)
Number of children	0.0052	0.0107
	(0.0052)	(0.0074)
_cons	0.302***	0.0778***
	(0.0235)	(0.0116)
N	10,415	10,415
Kleibergen-Paap rk LM	26.451	27.559
Cragg-Donald Wald F	28.541	29.786
DWH test	5.179**	3.712***
First-stage F-statistic	2.871	2.786

① ***, **, and * signify that the estimation findings are significant at the 1, 5, and 10% levels, respectively. ② The robust standard errors are enclosed in parentheses.

### 4.3 Heterogeneity analysis

#### 4.3.1 Differences in entrepreneurship types

Considering the heterogeneity of entrepreneurship types, this paper further explores the impact of the improvement of gender role attitude on different types of entrepreneurships among rural mothers. Table 9 reports the estimation results: Models (1) and (2) show that the improvement of gender role attitude increases the probability of rural mothers engaging in subsistence entrepreneurship by 5.57%, while the increase in the probability of opportunity entrepreneurship is only 1.03%. Models (3) and (4) indicate that the improvement of gender role attitude raises the probability of rural mothers participating in subsistence entrepreneurship by 5.77%, whereas the increase in the probability of opportunity entrepreneurship is merely 1.12%.

**Table 9:** Differences in rural mothers’ entrepreneurship types.

Variables	(1)	(2)	(3)	(4)
	Necessity-based entrepreneurship	Opportunity-based entrepreneurship	Necessity-based entrepreneurship	Opportunity-based entrepreneurship
GRAS	0.0557***	0.0103***	0.0577***	0.0112***
	(0.0076)	(0.0034)	(0.0081)	(0.0032)
Age			−0.0061***	−0.0015***
			(0.0019)	(0.0004)
Religion			0.1635***	0.0186
			(0.0542)	(0.0126)
Years of education			0.0237***	0.0044***
			(0.0056)	(0.0011)
Household income			0.0069**	0.0117***
			(0.0028)	(0.0009)
Political identity			−0.2476**	−0.0384
			(0.1024)	(0.0244)
Health			0.0655***	0.0041
			(0.0176)	(0.0036)
Real Estate			0.0196	0.0086
			(0.0348)	(0.0082)
Car			0.1286***	0.0806***
			(0.0446)	(0.0151)
Number of children			0.0273	0.0021
			(0.0241)	(0.0053)
_cons	1.7126***	1.1186***	1.8194***	1.0106***
	(0.0551)	(0.0457)	(0.1412)	(0.0280)
Region	Yes	Yes	Yes	Yes
Year	Yes	Yes	Yes	Yes
N	10,163	5,395	10,163	5,395
*Pseudo R2*	0.029	0.031	0.049	0.042

① ***, **, and * signify that the estimation findings are significant at the 1, 5, and 10% levels, respectively. ② The robust standard errors are enclosed in parentheses.

Overall, the impact of the improvement of gender role attitude on rural mothers’ subsistence entrepreneurship is approximately 4% higher than that on their opportunity entrepreneurship. This indicates that the improvement of gender role attitude is more likely to promote rural mothers to engage in subsistence entrepreneurship, while its impact on opportunity entrepreneurship is relatively smaller. Subsistence entrepreneurship is more driven by livelihood pressure. After breaking free from the constraints of traditional gender roles, rural mothers are more inclined to alleviate income shortages through low-threshold entrepreneurial activities. In contrast, opportunity entrepreneurship relies on entrepreneurs’ market insight, business interest, and resource integration capabilities. Such entrepreneurial behavior requires more systematic knowledge reserves and entrepreneurial experience. However, limited by the resource endowment constraints in rural areas, rural mothers still face obstacles in the links of opportunity identification and development.

#### 4.3.2 Differences in education levels

Education level largely determines the cognitive ability of rural mothers and influences their professional skills and learning capabilities. Davidsson and Honig (2003) argued that the essence of education is a process of human capital accumulation, and farmers with a higher education level are more likely to engage in entrepreneurship. Considering the fundamental role of education in rural mothers’ entrepreneurship, this paper further explores the impact of gender role attitude on the entrepreneurship of rural mothers with different education levels. We divided rural mothers into three groups based on their education levels: incomplete compulsory education, secondary vocational education, and higher education.

Table 10 shows that the improvement of gender role attitude exerts heterogeneous impacts on the entrepreneurship of rural mothers with different educational levels. Models (1) and (2) indicate that the coefficients of gender role attitude are 0.0498 and 0.0347, respectively, both passing the significance test. This implies that among rural mothers who have not completed compulsory education and those with secondary education, the enhancement of gender role attitude promotes their entrepreneurial activities. Model (3) reveals that the coefficient of gender role attitude is −0.0569, but it fails to pass the significance test. This may be due to the diversified employment opportunities brought by higher education, which significantly reduces the necessity of entrepreneurship as an alternative means of livelihood. Consequently, their entrepreneurial behavior is more driven by opportunity rather than survival.

**Table 10:** Heterogeneity in rural mothers’ education levels.

Variables	(1)	(2)	(3)
	Failure to complete compulsory education	Secondary vocational education	Higher education
GRAS	0.0498***	0.0347***	−0.0569
	(0.00826)	(0.00759)	(0.0438)
Age	−0.00818***	−0.00631***	0.0203*
	(0.00205)	(0.00188)	(0.0118)
Religion	0.142**	0.133**	−0.1491***
	(0.0558)	(0.0526)	(0.0461)
Years of education	0.0323***	0.0274***	0.115
	(0.00685)	(0.00545)	(0.189)
Household income	0.0128***	0.0119***	0.0181*
	(0.0035)	(0.0027)	(0.0104)
Political identity	−0.0258	−0.292***	−0.618**
	(0.131)	(0.0984)	(0.274)
Health	0.0727***	0.0770***	−0.0275
	(0.0180)	(0.0169)	(0.119)
Real Estate	0.0214	0.0193	−0.155
	(0.0375)	(0.0330)	(0.120)
Car	0.218***	0.200***	0.288
	(0.0462)	(0.0412)	(0.217)
Number of children	0.0274	0.0215	−0.119
	(0.0246)	(0.0232)	(0.166)
_cons	1.843***	1.846***	2.852
	(0.149)	(0.134)	(3.047)
Region	Yes	Yes	Yes
Year	Yes	Yes	Yes
N	9,158	10,415	280
*Pseudo R2*	0.039	0.064	0.053

① ***, **, and * signify that the estimation findings are significant at the 1, 5, and 10% levels, respectively. ② The robust standard errors are enclosed in parentheses.

#### 4.3.3 Differences in entrepreneurial environments

The entrepreneurial environment determines entrepreneurial opportunities and market conditions, and reflects the external dependence of rural mothers’ entrepreneurship. Minniti (2010) found in his research that there is a linear relationship between GDP and women’s entrepreneurship. Considering the impact of the entrepreneurial environment on rural mothers’ entrepreneurial decisions, we divided the samples into eastern, central, and western regions according to China’s regional economic development gradient. Among them, the eastern region has a relatively better economy, followed by the central region, and the western region has a relatively weaker economy.

Table 11 shows that gender role attitude has a differentiated impact on the entrepreneurial decisions of rural mothers in different regions. Models (1) and (3) indicate that in China’s eastern and western regions, the improvement of gender role attitude significantly promotes rural mothers’ entrepreneurship. In contrast, Model (2) shows that the improvement of gender role attitude has a positive correlation with rural mothers’ entrepreneurship in the central region, but this correlation fails to pass the significance test. A further comparison of regional impact coefficients reveals that the driving effect of gender role attitude improvement on rural mothers’ entrepreneurship in the eastern region is significantly stronger than that in the central and western regions. This further verifies that the entrepreneurial environment determines entrepreneurial opportunities and market conditions, and a better economic environment is more conducive to rural mothers’ entrepreneurship.

**Table 11:** Differences in rural mothers’ entrepreneurial environments.

Variables	(1)	(2)	(3)
	Eastern	Central	Western
GRAS	0.0441***	0.0365	0.0131***
	(0.0131)	(0.0325)	(0.0013)
Age	−0.0028	−0.0096***	−0.0059*
	(0.0034)	(0.0031)	(0.0034)
Religion	0.2543***	0.0508	0.0433
	(0.0829)	(0.103)	(0.0939)
Years of education	0.0359***	0.0181**	0.0335***
	(0.0099)	(0.0089)	(0.0099)
Household income	0.0012**	0.0111***	0.0159**
	(0.0044)	(0.0042)	(0.0065)
Political identity	−0.6335***	−0.2174	−0.1434
	(0.2132)	(0.1612)	(0.1573)
Health	0.1194***	0.0637**	0.0482
	(0.0298)	(0.0286)	(0.0297)
Real Estate	−0.0358	0.1033	0.0127
	(0.0548)	(0.0639)	(0.0561)
Car	0.2445***	0.2916***	0.0345
	(0.0712)	(0.0665)	(0 0.0795)
Number of children	0.0535	0.0165	0.0174
	(0.0411)	(0.0407)	(0.0401)
_cons	2.148***	1.746***	1.762***
	(0.245)	(0.226)	(0.237)
Region	Yes	Yes	Yes
Year	Yes	Yes	Yes
*N*	3,389	3,971	3,055
R2	0.0494	0.0468	0.0332

① ***, **, and * signify that the estimation findings are significant at the 1, 5, and 10% levels, respectively. ② The robust standard errors are enclosed in parentheses.

## 5 Mechanism analysis

### 5.1 The importance of family gender attitudes

In China’s traditional Confucian ideological system, the values of “male superiority over females” and “obedience to fathers and husbands” require women to follow the arrangements of their fathers and husbands. Therefore, the gender attitudes of fathers and husbands directly shape women’s self-perception. If a father or husband holds traditional attitudes, women tend to be confined to the family division of labor model where “men work outside the home and women take care of household affairs.” On the contrary, if a father or husband holds gender-equal attitudes, women are more likely to break gender biases and release their economic potential. Considering the importance of husbands and fathers, this paper further explores the impact of family attitudes on rural mothers’ entrepreneurship. Generally speaking, there is a significant positive correlation between education level and gender attitudes. Therefore, we use the husband’s education level and the father’s education level as proxy variables for family attitudes to measure the impact of family attitudes on rural mothers’ entrepreneurship.

Table 12 reports the regression results of family gender attitudes. Models (1) and (2) show that the regression coefficient of the interaction term between the husband’s education level and gender role attitude is 0.0418, and the regression coefficient of the interaction term between the father’s education level and gender role attitude is 0.0117, both passing the significance test. This indicates that the higher the education level of male family members, the stronger the positive moderating effect of gender role attitude on rural mothers’ entrepreneurship. This result verifies our Hypothesis 2 and confirms that family support, especially that from husbands and fathers, plays an important role in rural mothers’ entrepreneurship.

**Table 12:** The power of family in rural mothers’ entrepreneurship.

Variables	(1)	(2)
	Husband’s educational background	Father’s educational background
GRAS	0.0807***	0.0527***
	(0.0221)	(0.0117)
Spouse Education	0.0324*	
	(0.0176)	
Spouse*GRAS	0.0418***	
	(0.0138)	
Father Education		0.0239*
		(0.0133)
Father*GRAS		0.0117**
		(0.0058)
Age	−0.0063***	−0.0051**
	(0.0019)	(0.0021)
Religion	0.1345**	0.1317**
	(0.0524)	(0.0525)
Years of education	0.0261***	0.0243***
	(0.0058)	(0.0056)
Household income	0.0119***	0.0117***
	(0.0026)	(0.0026)
Political identity	−0.2853***	−0.2965***
	(0.0924)	(0.0924)
Health	0.0762***	0.0771***
	(0.0169)	(0.0169)
Real Estate	0.0194	0.0167
	(0.0322)	(0.0322)
Car	0.2012***	0.1934***
	(0.0412)	(0.0412)
Number of children	0.0216	0.0232
	(0.0225)	(0.0226)
_cons	2.110***	1.979***
	(0.198)	(0.151)
Region	Yes	Yes
Year	Yes	Yes
*N*	10,415	10,415
*Pseudo R2*	0.0414	0.0420

① ***, **, and * signify that the estimation findings are significant at the 1, 5, and 10% levels, respectively. ② The robust standard errors are enclosed in parentheses.

### 5.2 The importance of information

In traditional rural families, rural mothers mainly obtain information from within the family, which to a certain extent traps them in the dilemma of an “information cocoon.” The popularization of Internet technology has fundamentally restructured the paradigm of information dissemination. By breaking the information gap across space and time, it provides a new way for rural mothers to access information. On the one hand, the abundant information on the Internet helps rural mothers break the constraints of traditional concepts and form more positive and open gender attitudes; on the other hand, it enables rural mothers to keep abreast of changes in the external environment and entrepreneurial information. In addition, the typical entrepreneurial cases and successful experiences displayed on Internet platforms are more likely to stimulate rural mothers’ entrepreneurial interest and confidence. Considering the importance of information, this paper further explores the impact of information acquisition on rural mothers’ entrepreneurship.

Model (1) in Table 13 shows that the regression coefficient of the interaction term between information acquisition channels and gender attitudes is 0.0025, which has passed the significance test. This indicates that internet use exerts a positive moderating effect on the impact of gender role attitude on rural mothers’ entrepreneurship. This verifies Hypothesis 3 of this study: the openness of information acquisition enables rural mothers to break through the constraints of traditional gender role attitude and increase their probability of engaging in entrepreneurship.

**Table 13:** The power of information and the spillover effect.

Variables	(1)	(2)
GRAS	0.0334**	0.0717***
	(0.0162)	(0.0242)
Information	0.0311*	
	(0.0182)	
Information*GRAS	0.0025**	
	(0.0011)	
Employment attitude		0.0552**
		(0.0256)
Employment attitude*GRAS		0.0086**
		(0.0042)
Age	−0.0064***	−0.0063***
	(0.0019)	(0.0019)
Religion	0.1325**	0.1335**
	(0.0524)	(0.0524)
Years of education	0.0279***	0.0274***
	(0.0054)	(0.0054)
Household income	0.0012***	0.0012***
	(0.0002)	(0.0002)
Political identity	−0.2935***	−0.2906***
	(0.0923)	(0.0924)
Health	0.0769***	0.0772***
	(0.0169)	(0.0169)
Real Estate	0.0188	0.0196
	(0.0322)	(0.0321)
Car	0.2086***	0.2014***
	(0.0432)	(0.0412)
Number of children	0.0217	0.0216
	(0.0225)	(0.0225)
_cons	1.708***	2.014***
	(0.295)	(0.196)
Region	Yes	Yes
Year	Yes	Yes
N	10,415	10,415
*Pseudo R^2^*	0.0419	0.0411

① ***, **, and * signify that the estimation findings are significant at the 1, 5, and 10% levels, respectively. ② The robust standard errors are enclosed in parentheses.

### 5.3 The spillover effect of entrepreneurship

Women’s entrepreneurship has a significant spillover effect on promoting gender equality in the workplace. Existing studies have shown that female entrepreneurs pay special attention to the rights and interests of female employees and strive to break the restrictions and prejudices against women in the traditional workplace. In addition, female entrepreneurs also attach great importance to the issue of gender equality in the workplace and take corresponding measures to advance this process. Considering the spillover effect of women’s entrepreneurship, this paper further explores the spillover effect of rural mothers’ entrepreneurship.

Model (2) in Table 13 shows that the regression coefficient of the interaction term between attitudes toward women’s employment and gender role attitude is 0.0086, which has passed the significance test. This indicates that the entrepreneurial activities of rural mothers provide more employment opportunities for women. Moreover, even during economic downturns, female entrepreneurs tend to exercise caution in deciding whether to lay off female employees. Thus, women’s entrepreneurship generates a spillover effect, thereby benefiting female workers.

## 6 Discussion

Whether the improvement of gender role attitude has reshaped women’s entrepreneurship is of great practical significance for releasing women’s economic potential and promoting inclusive economic development. This paper uses the Chinese General Social Survey to analyze the impact of the improvement of gender role attitude on rural mothers’ entrepreneurship.

The study shows that the improvement of gender role attitude has a significant promoting effect on rural mothers’ entrepreneurship. This conclusion verifies the views of many scholars, who argue that gender discrimination exists in developing countries, and that as gender attitudes improve, the possibility of women’s entrepreneurship will increase (Acs et al., 2011; Bahramitash and Salehi Esfahani, 2014; Feldmann et al., 2022). For example, Kuada (2009) believes that gender equality is an important symbol of social progress, and when women realize that they also have entrepreneurial capabilities, they will carry out their own businesses more actively. Liñán et al. (2022) found that under economic pressure, mothers are more inclined to seek economic income through entrepreneurship to improve family life and provide better education and living conditions for their children.

Although the research conclusions of this paper have verified the views of some scholars to a certain extent, it is undeniable that not all scholars hold the same opinion. Some scholars argue that rural women who take on the role of mothers face a more severe “motherhood penalty” in balancing family and career, which in turn forces them to return to the family (Du, 2023; Looze, 2014; Yu and Kuo, 2017).

In addition, other scholars believe that there are still economic, social and cultural barriers in rural areas, which manifest as gender gaps, regional gaps and digital gaps. These gaps increase the possibility of entrepreneurial failure for rural women (Amine and Staub, 2009; Antonio and Tuffley, 2014; Omotoso et al., 2020). It is important to note that these studies have not fully considered factors such as women’s active learning ability, the breadth of information dissemination, and the improvement of gender attitudes driven by political and social efforts (Shao et al., 2023). In fact, these factors are all important forces that promote the improvement of gender attitudes and the development of women’s entrepreneurship. Based on the CGSS database, we have incorporated more of these forces as control variables in our empirical analysis, which has made our conclusions more accurate.

Heterogeneity analysis shows that the improvement of gender attitudes exerts a stronger promotive effect on the survival-driven entrepreneurship of rural mothers, with the magnitude of this effect being approximately 4% higher than that on opportunity-driven entrepreneurship. Constrained by the resource endowments and ideological perceptions in rural areas, rural mothers usually choose survival-driven entrepreneurship to address the issue of insufficient income. However, opportunity-driven entrepreneurship largely depends on entrepreneurs’ market acumen and resource integration capabilities, which rural mothers generally lack.

Furthermore, our study has also found some interesting conclusions. The empirical results show that rural mothers with higher education have a weaker entrepreneurial awareness, which contradicts the conclusion that “education promotes entrepreneurship.” However, this view has also been verified by Williams et al. (2015) and Datzberger (2018). There are two reasons for this result. First, rural mothers with higher education are more inclined to choose a stable career path rather than high-risk entrepreneurship. Second, there is a disconnect between higher education and entrepreneurial skills. This reminds us that in the process of supporting women’s entrepreneurship, relying solely on the role of traditional education is insufficient, and it is also necessary to strengthen the training of entrepreneurial skills (Carter, 2000).

In the mechanism analysis, we found that family attitudes play an important role in promoting rural mothers’ entrepreneurship. In the context of the widespread patriarchal ideology in rural areas, encouragement from husbands and fathers helps rural mothers to start their own businesses. This is because men possess more resources and discourse power, and can provide support in terms of funds, labor, and social capital, thereby reducing entrepreneurial risks (Dal Mas and Paoloni, 2019; Little, 1987). On the other hand, information dissemination not only improves rural mothers’ gender attitudes, thereby promoting their entrepreneurship, but also provides them with more entrepreneurial opportunities. A study by Shao et al. (2022) shows that internet use can increase the entrepreneurial probability of rural mothers by 7.88%. In addition, we also found that there is a positive spillover effect in rural mothers’ entrepreneurship, that is, rural mothers’ entrepreneurship provides more employment opportunities for other women.

## 7 Research limitations

Although our study has made some new contributions in terms of the perspective on women’s entrepreneurship and its transmission mechanism, there are still some shortcomings, which may provide new research directions for other scholars and researchers. First, due to limited data, we only analyzed the entrepreneurial behavior of rural mothers, but did not discuss the performance or scale effect of their entrepreneurship. Second, quantifying gender attitudes is not a simple task and requires a more scientific evaluation index. Finally, although women’s entrepreneurship, as an emerging force for economic growth, will remain a hot topic in the future, the academic community still lacks a clear theoretical explanation for the research on rural mothers’ entrepreneurship. Therefore, there is still room for expansion in terms of extending the theoretical perspective, research scope, and research methods of women’s entrepreneurship. Continuing to expand the research scope of women’s entrepreneurship remains one of the important directions in the field of women’s entrepreneurship.

## 8 Research conclusions

Women’s entrepreneurship is of great practical significance for releasing women’s economic potential and promoting inclusive economic development. Over the past few decades, the scale and influence of women’s entrepreneurship have continued to rise, making it an important force driving social and economic transformation. However, in rural areas, female entrepreneurs face many challenges and constraints due to traditional gender stereotypes. With the deepening of China’s reform and opening - up, the gender attitudes of rural mothers have undergone tremendous changes. Against this background, whether the changes in gender attitudes have reshaped the entrepreneurial behavior of rural mothers is of great practical significance for alleviating poverty and promoting the inclusive development of the rural economy.

Based on the data from the Chinese General Social Survey (CGSS), this paper analyzes the impact of gender attitude improvement on rural mothers’ entrepreneurship and its transmission mechanism from both theoretical and empirical perspectives. Through the benchmark regression model, we find that the improvement of gender attitudes increases the entrepreneurship rate of rural mothers by 1.61%.

To ensure the robustness of the research results, we use methods such as the instrumental variable approach, alternative regression methods, and exclusion of outliers to verify the stability of the empirical results. The heterogeneity analysis shows that the impact of gender attitude improvement on the necessity-based entrepreneurship of rural mothers is 5.9% higher than that on opportunity-based entrepreneurship. The improvement of gender attitudes has a significant promoting effect on the entrepreneurship of rural mothers who have not completed higher education, but shows the opposite effect on those who have completed higher education.

In terms of differences in entrepreneurial environments, a better economic environment is more conducive to the entrepreneurship of rural mothers. The mechanism analysis reveals that family support, especially support from husbands and fathers, plays a crucial role in rural mothers’ entrepreneurship. The openness of information acquisition enables rural mothers to break through the constraints of traditional gender concepts and increases their probability of starting a business. In addition, our study also finds that the entrepreneurial behavior of rural mothers has a spillover effect, that is, rural mothers provide more employment opportunities for other women through their entrepreneurial activities.

Based on the above conclusions, this study puts forward the following policy recommendations: First, establish a protection mechanism for the economic rights and interests of rural women to strengthen their economic autonomy. Second, promote gender equality attitudes through channels such as radio, television, and the Internet. Third, continuously advance the popularization of compulsory education in rural areas to improve the basic cultural literacy of women from the source. Fourth, accelerate the popularization of the Internet in rural areas to break down information barriers. Fifth, conduct customized entrepreneurial training to enhance skill support.

## Data Availability

The original contributions presented in the study are included in the article/supplementary material, further inquiries can be directed to the corresponding author.
